# Triptolide-induced cuproptosis is a novel antitumor strategy for the treatment of cervical cancer

**DOI:** 10.1186/s11658-024-00623-4

**Published:** 2024-08-28

**Authors:** Yanxia Xiao, Jiameng Yin, Pu Liu, Xin Zhang, Yajun Lin, Jun Guo

**Affiliations:** The Key Laboratory of Geriatrics, Beijing Institute of Geriatrics, Institute of Geriatric Medicine, Chinese Academy of Medical Sciences, Beijing Hospital/National Center of Gerontology of National Health Commission, NO.1 Da HuaRoad, DongDan, Beijing, 100730 People’s Republic of China

**Keywords:** Cuproptosis, Triptolide, XIAP, COMMD1, Cervical cancer

## Abstract

**Background:**

Cuproptosis is a unique copper-dependent form of cell death that is highly correlated with the metabolic state of cells. Triptolide exerts pharmacological activity by altering the regulation of metal ions. Cuproptosis is poorly understood in cancer, so in this study, we explored whether triptolide could induce cuproptosis in cervical cancer cells.

**Methods:**

The human cervical cancer cell lines HeLa and SiHa, which primarily rely on oxidative phosphorylation, were treated with triptolide. Cell viability, proliferation and migration, copper levels and cuproptosis-related protein levels were evaluated in these cell lines. The copper ion chelator tetrathiomolybdate (TTM) was administered to determine whether it could reverse the cuproptosis induced by triptolide. In addition, a nude mouse cervical cancer xenograft model was established to determine the effects of triptolide on cuproptosis in isolated tumor tissues.

**Results:**

The copper concentration increased with triptolide treatment. The levels of cuproptosis -related proteins, such as FDX1, LIAS, and DLAT, in the HeLa and SiHa cell lines decreased with triptolide treatment. XIAP, the target of triptolide, played a role in cuproptosis by regulating COMMD1. The level of copper exporters (ATP7A/B) decreased, but the level of the copper importer (CTR1) did not change with triptolide treatment. Furthermore, triptolide inhibited cervical cancer growth and induced cuproptosis in vivo.

**Conclusions:**

In summary, we report a new antitumor mechanism by which triptolide disrupted intracellular copper homeostasis and induced cuproptosis in cervical cancer by regulating the XIAP/COMMD1/ATP7A/B axis.

**Supplementary Information:**

The online version contains supplementary material available at 10.1186/s11658-024-00623-4.

## Background

An increasing amount of evidence shows that imbalances in the metabolism of intracellular heavy metals, such as iron [1], zinc [2], manganese [3], calcium [4], and copper [5], promote cell death. In a recent study, Tsvetkov et al. proposed a novel form of copper-induced cell death, namely, cuproptosis. Cuproptosis is a unique type of copper-dependent cell death that, unlike other existing forms of cell death, is driven by intracellular copper overload. This process can be summarized as follows: excessive free copper in cells forms polymers by directly binding lipoylated proteins in the tricarboxylic acid cycle and leads to the loss of iron-sulfur cluster proteins, ultimately leading to cell death [5].

Triptolide (TPL) is a diterpenoid triepoxide that is isolated from the root of *Tripterygium wilfordii* and has significant antitumor, immunosuppressive and anti-inflammatory effects [6]. TPL has been shown to have an antitumor impact on breast cancer, ovarian cancer, endometrial cancer, pancreatic cancer and other malignant tumors [7–9]. Triptolide exerts pharmacological effects by altering the metabolism of various metal ions, such as iron and calcium, in cells [10, 11]. Moreover, Triptolide can improve the neuroinflammation and behavioral defects of multiple sclerosis model induced by copper chelating agent Cuprizone [12]. However, whether triptolide affects intracellular copper metabolism and thus plays an antitumor role remains unclear.

Cuproptosis is related to cellular metabolism and is more likely to occur in cell lines and tumors that rely on mitochondrial metabolism [5, 13]. HeLa and SiHa cells rely primarily on oxidative phosphorylation/mitochondrial metabolism, so exploring the cuproptosis pathway in these two cervical cancer cell lines is feasible [14]. Cervical cancer is the fourth most prevalent gynecological malignancy in the world and poses a serious threat to women's health. Additionally, one of the main causes of cancer-related deaths in women is cervical cancer [15]. Among young adults in the United States, the incidence of cervical cancer (aged 30–44) is increasing 1–2% every year. Cervical cancer has always been the second leading cause of cancer death among women aged 20–39 [16]. Therefore, there is an urgent need to find new treatment modalities to provide hope to patients. In this study, we examined whether triptolide can exert antitumor effects in cervical cancer through cuproptosis both in vivo and in vitro and explored its role in cuproptosis.

## Methods

### Reagents and chemicals

Thiazolyl blue tetrazolium bromide (MTT) (M8180) was purchased from Solarbio (Beijing, China). Triptolide (HY-32735, 99.8% purity), ferrostatin-1 (Fer-1) (HY-100579), Z-VAD (OMe)-FMK (Z-VAD) (HY-16658), 3-methyladenine (3-MA) (HY-19312), necrostatin-1 (Nec-1) (HY-15760), acetylcysteine (NAC) (HY-B0215), and JC-1 (HY-15534) were obtained from MedChemExpress (Shanghai, China). Triptolide was dissolved in dimethyl sulfoxide to make a stock solution, which was stored at − 80 °C. A Cu^2+^ fluorescent probe (JY0299) was purchased from BIOFOUNT (Beijing, China). Dimethyl sulfoxide (DMSO), stripping buffer (P0025), and the BeyoClick™ EdU Cell Proliferation Kit with Alexa Fluor 488 (C0071S) were purchased from Beyotime Biotechnology (Shanghai, China). A reduced glutathione (GSH) assay kit (A006-2-1) was purchased from Nanjing Jiancheng Institute of Biological Engineering (Nanjing, China). Tetrathiomolybdate (TTM) (323446) was purchased from Sigma Aldrich (Darmstadt, Germany). The primary antibodies FDX1 (ab108257), POLD1 (ab186407), ACO-2 (ab129069), SDHB (ab178423), and CTR1 (ab129067) were purchased from Abcam (Shanghai, China). Lipoic acid (cat# 437695) was obtained from Millipore. ATP7A (sc-376467) and ATP7B (sc-373964) were obtained from Santa Cruz Biotechnology (Santa Cruz, CA, USA). DLAT (12362), XIAP (2042), β-Actin (4970) and GAPDH (5174) were purchased from Cell Signaling Technology (Beverly, MA, USA). LIAS (11577-1-AP) and vinculin (66305-1-Ig) were purchased from Proteintech (Wuhan, China). COMMD1 (H00150684-M01) was obtained from Abnova. The secondary antibodies goat anti-rabbit IgG (ZB-2301) and goat anti-mouse IgG (ZB-2305 and ZF-0312) were purchased from Beijing Zhong Shan-Golden Bridge Biological Technology (Beijing, China).

### Cell lines

The human cervical carcinoma cell lines HeLa (ATCC CCL-2) and SiHa (ATCC HTB-35) were purchased from the American Tissue Culture Collection (ATCC).

### Cell viability assays

Cell viability was assessed using MTT assays. A total of 3 × 10^3^ HeLa or SiHa cells per well were added to 96-well plates. Then, triptolide (20, 40, 80, and 160 nmol/l) was applied to the cells for 48 h, 5 mg/ml MTT solution (20 µl/well) was added, and the cells were incubated for 4 h. To dissolve the formazan crystals, 150 µl of DMSO was added after the supernatants were removed. The optical density (OD) was determined using a microplate reader (Multiskan™ MK3; Thermo Fisher Scientific, Inc., US) at a wavelength of 490 nm. The half maximal inhibitory concentration (IC_50_) values were calculated using GraphPad Prism 9.0 software (GraphPad Software, Inc.). In the chemical rescue experiments, Fer-1, Z-VAD-FMK, 3-MA, Nec-1, and NAC were added 2 h before the addition of triptolide. Cell viability was measured 48 h after the addition of drugs.

### EdU cell proliferation assay

The BeyoClick™ EdU Cell Proliferation Kit with Alexa Fluor 488 was used to assess cell proliferation according to the manufacturer's instructions. A fluorescence microscope (BZ-X810, Keyence) was ultimately used to capture the images.

### Cell migration assay

A 24-well transwell chamber system (Corning, 3422) was used to conduct the cell migration assay. The Transwell apparatus was separated into the upper and lower compartments by a special filter. In the upper compartment, HeLa and SiHa cells were suspended in 200 μL of growth medium. Then, 600 μL of growth medium supplemented with 10% fetal bovine serum (FBS) was added to the lower chamber. Triptolide was then added to the upper chamber. Cells on the upper filter surface were removed by wiping with a cotton swab following a 24-h incubation at 37 °C in an incubator. The filters were then fixed in 4% paraformaldehyde and stained with 0.4% crystal violet. The number of cells that migrated to the lower filter surface was recorded using a microscope (BZ-X810, Keyence**)**.

### Wound healing assay

In 6-well plates, HeLa and SiHa cells were plated until a monolayer developed. A line was scratched using a 200 µl pipette tip. The cells were washed 3 times with PBS to remove suspended cells, growth medium supplemented with 2% FBS was added to the 6-well plates, and the scratch data were recorded using a microscope (BZ-X810, Keyence**)**. Then, the cells were treated with triptolide and cultured in an incubator for 48 h. The suspended cells were removed, and the scratch data were recorded using a microscope (BZ-X810, Keyence**)**. The wound healing region was examined using ImageJ software. The difference between the 0 h and 48 h areas was determined.

### GSH determination

The levels of glutathione (GSH) were measured using a reduced GSH assay kit according to the manufacturer’s instructions.

### Mitochondrial membrane potential

The mitochondrial membrane potential was examined using JC-1 staining. In a 6-well plate, 1 × 10^5^ HeLa and SiHa cells were cultured overnight. Then, the culture medium was replaced with fresh medium containing 80 nM or 40 nM TPL, followed by a 48-h incubation; then, the culture medium was discarded and the cells were treated with JC-1 working solution (prewarmed serum-free cell culture medium containing 10 μg/mL JC-1) for 20 min at 37 °C in the dark. The cells were examined under a fluorescence microscope (BZ-X810, Keyence) after being washed with serum-free cell culture media.

### Cu^2+^ fluorescence staining

HeLa and SiHa cells were treated with triptolide for 48 h. A Cu^2+^ fluorescence probe was added to the culture medium at a working concentration of 10 μM, and the HeLa and SiHa cells were incubated at 37 °C in the dark for 20 min. The dye working solution was removed, the cells were washed twice with growth medium, and the cells were observed under a fluorescence microscope (BZ-X810, Keyence).

### Immunofluorescence

HeLa and SiHa cells were fixed with 4% paraformaldehyde for 15 min. After treatment with 0.3% Triton X-100 for 15 min, the cells were blocked with 5% bovine serum albumin (A6020, Biotopped) for 30 min at room temperature. An anti-DLAT antibody (1:100, 12362, Cell Signaling) was used as the primary antibody for incubation. The secondary antibody utilized was FITC-conjugated goat anti-mouse IgG (ZF-0312). The nuclei were labeled with DAPI staining solution (Beyotime).

### Inductively coupled plasma‒mass spectrometry (ICP-MS)

The collected cell precipitate was resuspended in 1 ml of PBS, and the cell suspension was crushed with an ultrasonic crusher. One milliliter of 1% nitric acid was added to 1 ml of the crushed cell suspension, which was then incubated at 60 °C for one hour. Another 10 µl of the cell suspension was removed for protein quantification. The Cu content of the cell sample was measured by inductively coupled plasma‒mass spectrometry (ICP-MS, Perkin Elmer, Nexion 350, Waltham, MA, USA). The copper concentration was normalized to the protein concentration.

### Western blot analysis

The culture medium of cell samples was removed, and the cells were washed twice with PBS. After RIPA lysis buffer containing 1 mM PMSF was added, the cells were lysed on ice for 30 min, and the cell samples were collected with a cell scraper. For tissue samples, RIPA buffer was added to the tissue, which was subsequently ground, crushed via ultrasonication, and lysed on ice for 30 min. After centrifuging the samples at 12,000 rpm for 20 min at 4 °C, the pellet was removed, and Pierce™ BCA Protein Assay Kits (23227, Thermo Fisher Scientific) were used to measure the protein concentration in the supernatant. Loading buffer was added, and the mixture was heated at 95 °C for 10 min. Proteins were first separated by 8–12% SDS‒PAGE at 80 V for 30 min and then at 120 V for 1 h. After the electrophoresis was stopped, the protein was transferred to a 0.2 μm PVDF membrane, the membrane was blocked with 5% skim milk powder for 2 h at room temperature and incubated with the corresponding primary antibody overnight at 4^◦^C. Then, after washing with TBST, the corresponding HRP-conjugated secondary antibody was incubated with the membrane for 1 h at room temperature. Following TBST washes, specific protein bands were detected with Immobilon Western HRP Substrate (WBKLS0500, Millipore) and a chemiluminescence imaging system (FluorChem HD2, ProteinSimple or Tanon 4800, Tanon).

### Quantitative real-time polymerase chain reaction

Total RNA was extracted from HeLa and SiHa cells using TRIzol Reagent (15596018, Invitrogen). The RNA samples were subjected to quantitative PCR with the corresponding primers using the One Step SYBR® PrimeScript™ RT‒PCR Kit (RR066A, Takara) and the Thermal Cycler Dice Real Time System (Takara) according to the manufacturer’s instructions. β-actin and GAPDH were used as the internal controls. The results were analyzed using the 2^−ΔΔCT^ method.

In the experiments, the following primers were used: ATOX1, Forward, 5′-GTGCTGAAGCTGTCTCTCGG-3′, and Reverse 5′-GCCCAAGGTAGGAAACAGTCTTT-3′; COX17, Forward, 5′-TGCGTGTATCATCGAGAAAGGA-3′, and Reverse 5′-GCCTCAATTAGATGTCCACAGTG-3′; CCS, Forward, 5′-GGGGACCTTACAAACAACTGC-3′, and Reverse 5′-GCATCAGCACGGACATTGC-3′; ATP7A, Forward, 5′-CTGAAATCTATGGCCTTAGAAG-3′, and Reverse 5′-CATTGCTACCCGTTTCC-3′; ATP7B, Forward, 5′-CTTGGGATACTGCACGGACTTC-3′, and Reverse 5′-CCTCAGCCACTCACGGTTTC-3′; CTR1, Forward, 5′-TTGGCTTTAAGAATGTGGACCT-3′, and Reverse 5′-GACTTGTGACTTACGCAGCA-3′; β-actin, Forward, 5′-TGTGGCATCCACGAAACTAC-3′, and Reverse 5′-GGAGCAATGATCTTGATCTTCA-3′; and GAPDH, Forward, 5′-CTCTGACTTCAACAGCGAC-3′, and Reverse 5′-CGTTGTCATACCAGGAAATG-3′.

### Copper salt stain kit

Analysis was performed using a copper salt stain kit (G3040, Solarbio Life Science, China) according to the manufacturer’s instructions.

### Network pharmacology and protein‒ligand docking

The Traditional Chinese Medicine Systems Pharmacology Database and Analysis Platform (TCMSP) was used to retrieve the targets of triptolide. The GeneCards database (https://www.genecards.org/) was used to retrieve potential targets for cervical cancer and copper homeostasis. The three-dimensional structure of the XIAP protein (5oqw) was obtained from UniProt (https://www.uniprot.org/). The mol2 file of triptolide (MOL003187) was obtained from TCMSP. Protein‒ligand docking was carried out on the CB-Dock2 website (https://cadd.labshare.cn/cb-dock2/) [17].

### Nude xenograft mouse model

This study was approved by the Institutional Animal Care and Use Committee of the Institute of Medicinal Biotechnology, Chinese Academy of Medical Sciences. Six- to eight-week-old BALB/c male nude mice were purchased from Beijing Huafukang Biotechnology (Beijing, China). Mice were kept in conventional pathogen-free settings with simulated 12-h light/dark cycles and consistent humidity levels of 50 to 60%. Throughout the trial, the mice had free access to standard laboratory food and tap water. After seven days of adaptive rearing, 5 × 10^6^ SiHa cells in 100 μL of PBS were injected subcutaneously into the dorsal right axilla of nude mice. One week after injection, 24 mice were randomly divided into four groups: (1) blank control, (2) low-dose triptolide (0.2 mg/kg), (3) middle-dose triptolide (0.4 mg/kg), and (4) high-dose triptolide (0.6 mg/kg). All treatments were given every day for two weeks. Every two days, the body weights of mice were recorded, and the tumor volume was determined using the following formula: [ (length × width × width)/2]. After 15 days of treatment, the mice were euthanized. The tumor tissues and relevant organs were collected.

### Statistical analysis

All the statistical analyses were performed using GraphPad Prism 9.0. Two-tailed unpaired Student’s t tests were used for comparisons of two groups. One-way ANOVA was used to determine the statistical significance of multiple comparisons.* p* < 0.05 was considered to indicate statistical significance.

## Results

### Triptolide increased the concentration of copper in cervical cancer cells

**Fig. 1:**
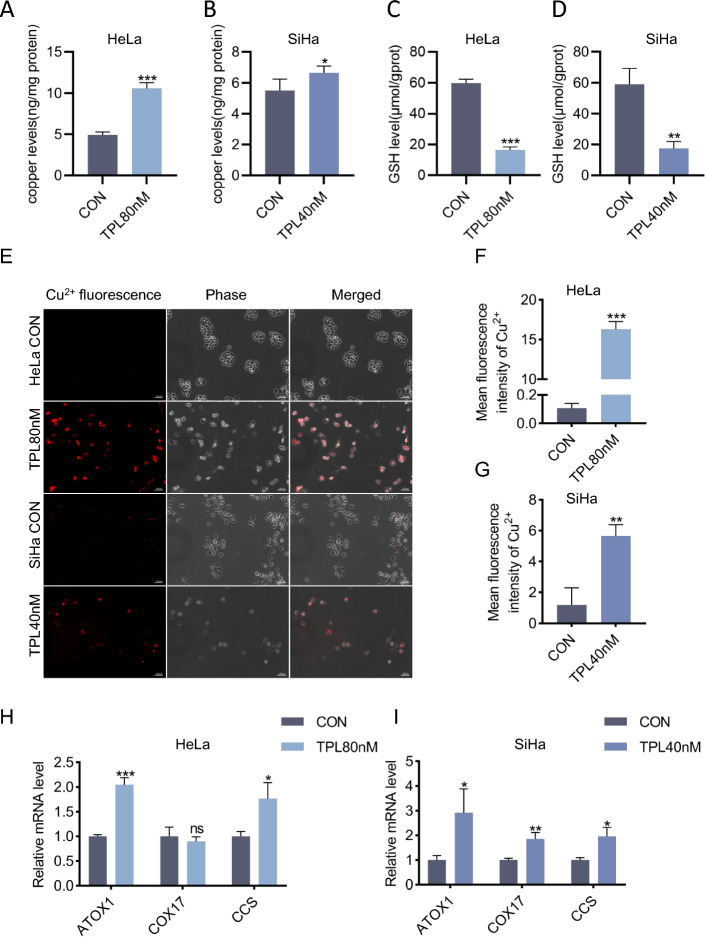
Triptolide increased the intracellular copper concentration in cervical cancer cells. **A**, **B** Copper levels were assessed by ICP‒MS in HeLa and SiHa cells treated with or without triptolide for 48 h. **C**, **D** GSH levels were assessed using a reduced glutathione assay in HeLa and SiHa cells following triptolide treatment for 48 h. **E** Representative images of Cu^2+^ fluorescence in HeLa and SiHa cells treated with or without triptolide for 48 h. Scale bars: 100 μm. **F**, **G** Mean fluorescence intensity of Cu^2+^ in HeLa and SiHa cells. **H**, **I** Relative mRNA levels of ATOX1, COX17 and CCS in HeLa and SiHa cells treated with or without triptolide for 48 h. (*p < 0.05, **p < 0.01, ***p < 0.001 versus the CON group.)

To investigate the ability of triptolide to induce cuproptosis, we investigated whether triptolide could increase the intracellular copper concentration. ICP‒MS was used to determine the intracellular copper concentration in cervical cancer cells treated with triptolide for 48 h. The results showed that the intracellular copper concentration of the two strains increased after triptolide treatment (Fig. 1A-B). GSH is a natural copper chaperone in cells, and an increase in the copper concentration reduces the GSH content in cells [18]. A reduced glutathione assay kit was used to determine the level of GSH in HeLa and SiHa cells after triptolide treatment for 48 h. The results showed that the level of GSH decreased significantly after triptolide treatment (Fig. 1C-D). Furthermore, copper ion fluorescence staining was used to determine the copper ion concentration in HeLa and SiHa cells. The results showed that the copper ion concentration in HeLa and SiHa cells increased obviously after 48 h of triptolide treatment (Fig. 1F-G). In the cytoplasm, copper can play a specific role by combining with chaperones such as ATOX1 (antioxidant 1 copper chaperone), CCS (copper chaperone for superoxide dismutase) and COX17 (cytochrome c oxidase copper chaperone 17) [19]. In our study, the mRNA expression levels of the copper chaperones ATOX1, CCS and COX17 increased in SiHa cells; the mRNA expression levels of ATOX1 and CCS increased in HeLa cells, but there was no significant change in COX17 expression in HeLa cells. Consistent with this result, the copper content in cancer tissues of many patients with solid tumors often increases, accompanied by upregulated expression of copper transport systems, such as ATOX 1 and CCS [20]. All the above results support the notion that triptolide can increase the concentration of copper in cervical cancer cells.

### Triptolide induces cuproptosis in cervical cancer cells

**Fig. 2:**
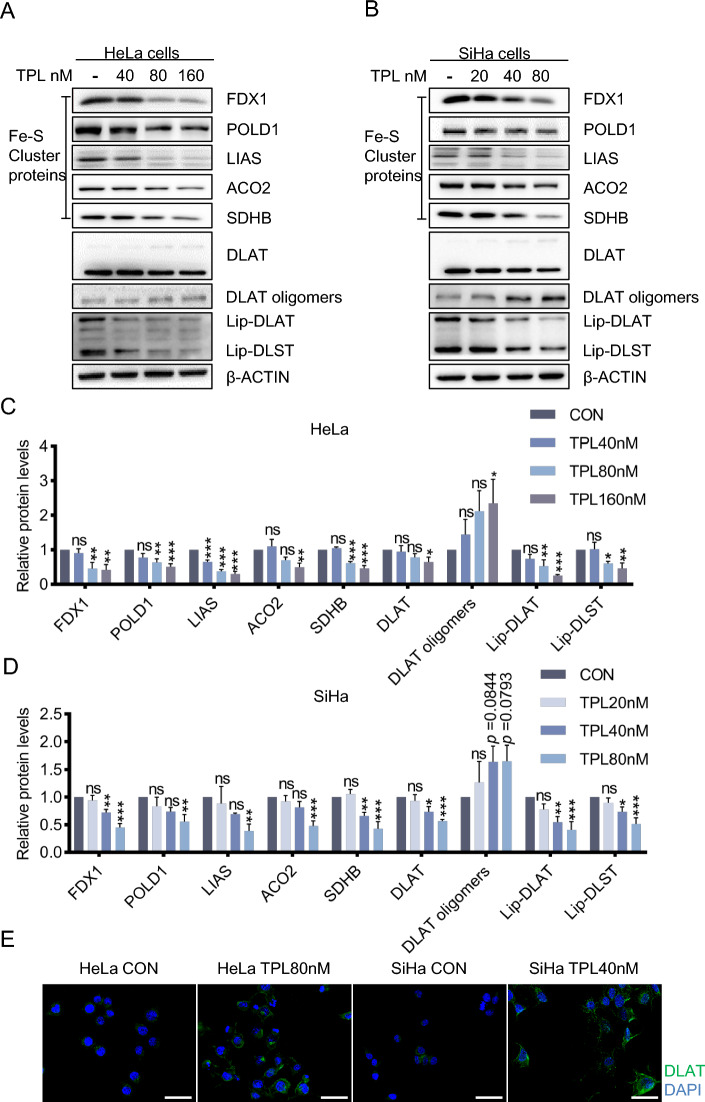
Triptolide induces cuproptosis in cervical cancer cells. **A** Western blotting of cuproptosis-related proteins in HeLa cells. **B** Western blot analysis of the levels of the apoptosis-related proteins in SiHa cells. **C** The protein content in HeLa cells was analyzed after triptolide treatment for 48 h. **D** The protein content in SiHa cells was analyzed after triptolide treatment for 48 h. **E** DLAT immunofluorescence after 80 nM or 40 nM triptolide treatment of HeLa and SiHa cells for 48 h (DALT-green, DAPI-blue). Scale bars: 50 μm. (*p < 0.05, **p < 0.01, ***p < 0.001 versus the CON group; ns, not significant.)

First, we verified the anticancer effect of triptolide in cervical cancer cells, and the results showed that triptolide inhibited the proliferation and migration of cervical cancer cells (Additional file 1: Fig. S1A-J). The IC_50_ values of triptolide in HeLa and SiHa cells at 48 h were 76.20 nM and 33.09 nM, respectively (Additional file 1: Fig. S1A-B). Second, the aggregation of lipoylated proteins and the loss of iron-sulfur cluster proteins are important signs of cuproptosis; thus, changes in these proteins were determined by western blotting in this study. With increasing triptolide concentration, the levels of DLAT and iron-sulfur cluster proteins gradually decreased, while that of DLAT oligomers gradually increased (Fig. 2A–D). In addition, through immunofluorescence experiments, we verified that the level of DLAT oligomer increased after treatment with triptolide (Fig. 2E). Finally, the main site of cuproptosis is the mitochondria, so morphological changes in the intracellular mitochondria before and after treatment with triptolide were observed via electron microscopy. After treatment with triptolide, the mitochondria of cells were obviously condensed, the matrix was concentrated, and the ridges were reduced and widened (Additional file 1: Fig. S3A). These results indicate that triptolide induces cuproptosis in cervical cancer cells.

### The copper ion chelator TTM can alleviate damage to cells caused by triptolide and cuproptosis induced by triptolide

**Fig. 3:**
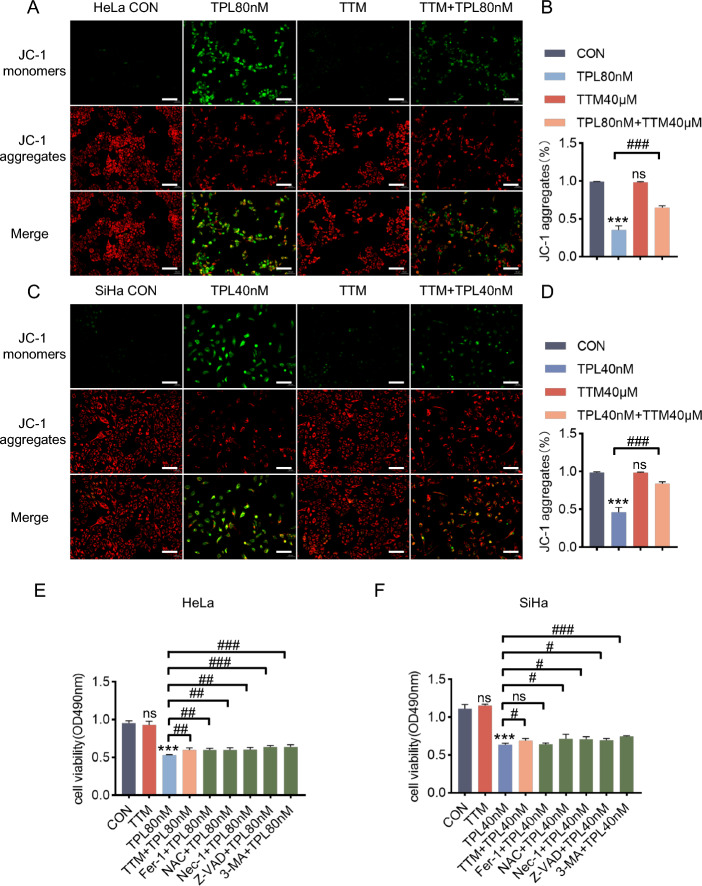
The copper ion chelator TTM can alleviate damage to cervical cancer cells caused by triptolide. **A** Representative photographs of JC-1-stained HeLa cells. HeLa cells were pretreated with or without TTM (40 μM) for 2 h, followed by treatment with or without triptolide for 48 h. Scale bars: 100 μm. **B** Measurement of the mitochondrial membrane potential of HeLa cells by JC-1 staining. **C** Representative images of JC-1-stained SiHa cells. SiHa cells were pretreated with or without TTM (40 μM) for 2 h and then treated with or without triptolide for 48 h. Scale bars: 100 μm. **D** Measurement of the mitochondrial membrane potential of SiHa cells by JC-1 staining. **E**, **F** HeLa and SiHa cells were preincubated with or without 40 μM TTM, 10 μM Fer-1, 10 μM NAC, 20 μM Nec-1, 20 μM 3-MA, or 20 μM Z-VAD for 2 h. Then, HeLa and SiHa cells were treated with 80 nM or 40 nM triptolide for 48 h. Cell viability was evaluated by MTT assay. (*p < 0.05, **p < 0.01, ***p < 0.001 versus the CON group; #*p* < 0.05, ##*p* < 0.01, ###*p* < 0.001 versus the TPL group; ns, not significant.)

**Fig. 4:**
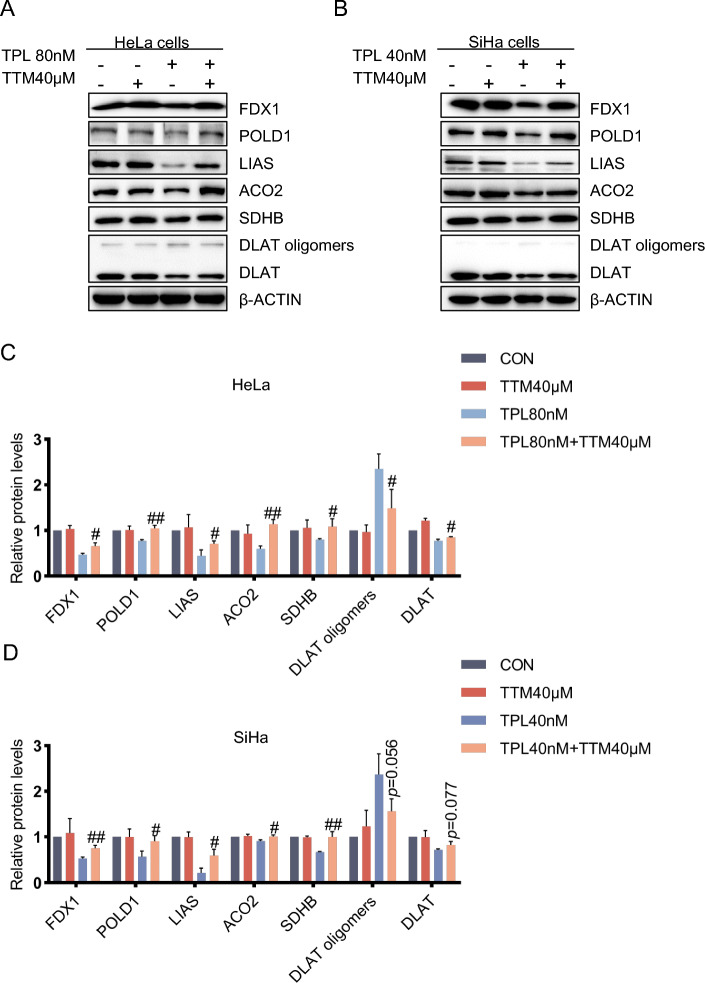
The copper ion chelator TTM alleviates triptolide-induced cuproptosis. **A**, **C** After pretreatment with or without 40 μM tetrathiomolybdate for 2 h, the protein content in HeLa cells was analyzed after treatment with 80 nM triptolide for 48 h. **B**, **D** After pretreatment with or without 40 μM TTM for 2 h, the protein content of SiHa cells was analyzed after 40 nM triptolide treatment for 48 h. It may be because the concentration of antibody is too high, which leads to the uneven background of LIAS in the lane. (#*p* < 0.05, ##*p* < 0.01, ###*p* < 0.001 versus the TPL group.)

Copper ion chelators can chelate copper ions to reduce the damage to cells caused by free copper. Therefore, in this study, the effect of the copper ion chelator TTM on cuproptosis induced by triptolide was investigated. First, with or without pretreatment with the copper chelator TTM, after 48 h of triptolide treatment, the mitochondrial membrane potential was determined via JC-1 staining (Fig. 3A, C). The results showed that the addition of the copper chelator TTM partially reversed damage to the mitochondrial membrane potential caused by triptolide (Fig. 3B, D). Second, after pretreatment with the copper ion chelator TTM and other inhibitors with known cell death mechanisms, MTT assays were used to determine the effects on triptolide-induced cervical cancer cell death. The results showed that TTM reduced cytotoxicity in HeLa and SiHa cells induced by triptolide (Fig. 3E-F) and confirmed the inhibitory effect of the copper chelator on cuproptosis induced by triptolide. Finally, the effect of the copper chelator TTM on the changes in the levels of cuproptosis-related proteins induced by triptolide was determined. The results showed that the copper chelator TTM could prevent cuproptosis induced by triptolide (Fig. 4A-B). The decrease in iron-sulfur cluster protein and DLAT levels in the TTM and triptolide combination group was less than that in the triptolide monotherapy group, and the increase in DLAT oligomer level in the combination group was less than that in the triptolide monotherapy group (Fig. 4C-D).

### Triptolide induces copper homeostasis imbalance by regulating the XIAP/COMMD1 pathway

**Fig. 5:**
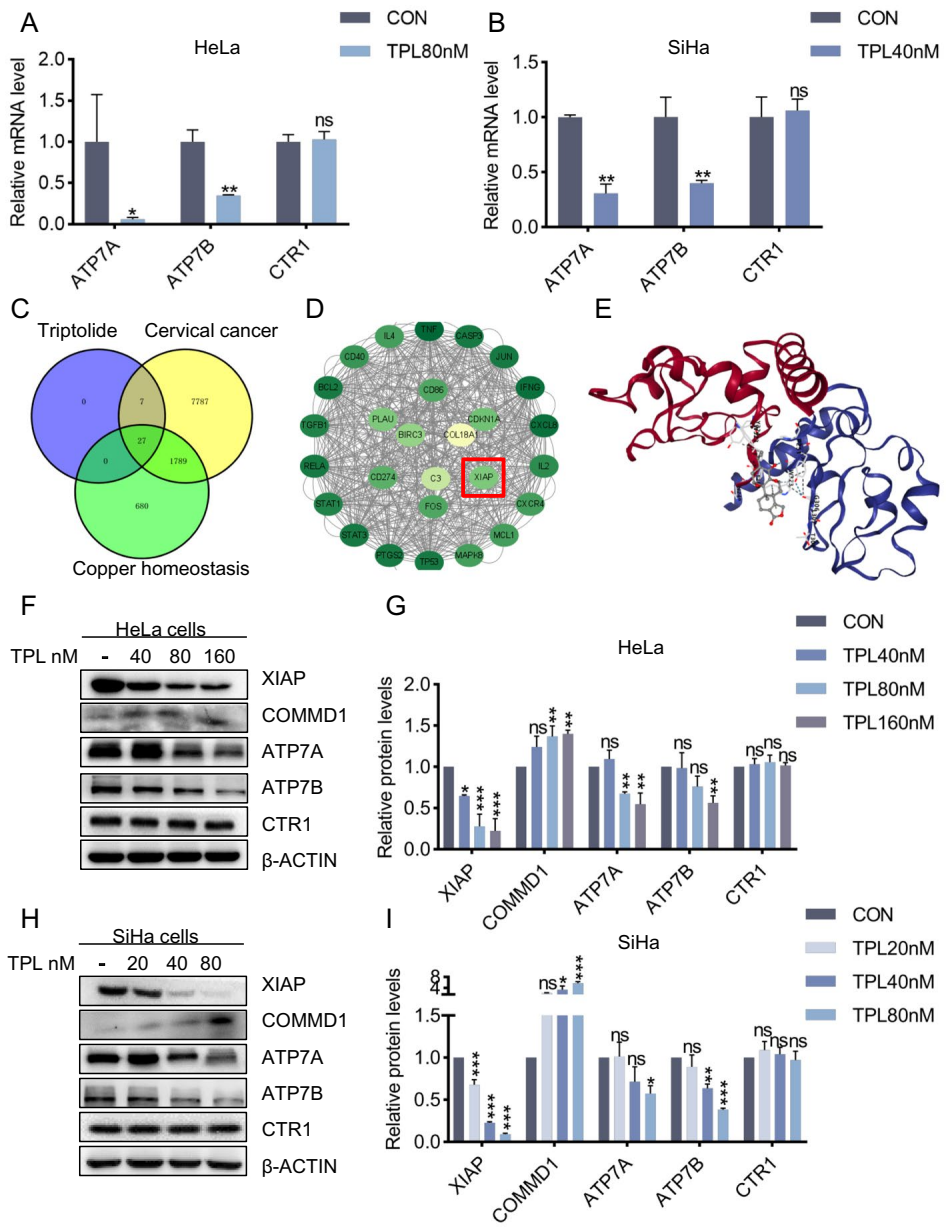
Triptolide induces copper homeostasis imbalance by regulating the XIAP/COMMD1 pathway. **A**, **B** Relative mRNA levels of ATP7A, ATP7B and CTR1 in HeLa and SiHa cells treated with or without triptolide for 48 h. **C** Venn diagram of triptolide, cervical cancer and copper homeostasis targets. **D** Visualization of 27 common targets. **E** Molecular docking between XIAP and triptolide. **F**, **G** The protein content of HeLa cells was analyzed after treatment with 80 nM triptolide for 48 h. **H**, **I** The protein content of SiHa cells was analyzed after 40 nM triptolide treatment for 48 h. (*p < 0.05, **p < 0.01, ***p < 0.001 versus the CON group; ns, not significant.)

The above results confirmed that triptolide can induce copper overload in cervical cancer cells and lead to cuproptosis. However, the specific mechanism by which triptolide regulates increases in the intracellular copper concentration is not clear. First, intracellular copper homeostasis is mainly maintained through copper importers (CTR1) and copper exporters (ATP7A/B). Under normal circumstances, the intracellular copper concentration is maintained at a low level [21]. Therefore, we investigated whether triptolide interferes with proteins that help maintain copper homeostasis by determining changes in the levels of CTR1 and ATP7A/B mRNA. The results showed that the level of CTR1 did not significantly change after triptolide treatment; notably, the mRNA expression levels of ATP7A and ATP7B decreased after triptolide treatment (Fig. 5A-B). We speculate that triptolide overloads intracellular copper by interfering with the regulation of copper homeostasis and subsequently induces cuproptosis in cervical cancer cells.

Starting with the reported targets of triptolide, we explored the relationship between triptolide and copper homeostasis and identified 27 common targets among triptolide, cervical cancer and copper homeostasis (Fig. 5C-D). A review of the literature revealed that X-linked inhibitor of apoptosis (XIAP) plays an important role in the regulation of copper homeostasis [22, 23]. XIAP accelerates copper excretion by acting as an E3 ubiquitin ligase of the copper metabolism gene MURR1-containing domain 1 (COMMD1) protein and promoting its proteasomal degradation [24]. COMMD1 regulates copper homeostasis by regulating the abundance of ATP7A and ATP7B [25, 26]. In addition, according to molecular docking, XIAP and triptolide tend to bind to each other (Fig. 5E) [17]. Triptolide is thought to regulate copper homeostasis by acting on the XIAP/COMMD1 pathway. Therefore, western blotting was used to determine the expression of XIAP, COMMD1, ATP7A and ATP7B in this pathway (Fig. 5F, H). The results showed that the expression of XIAP decreased, the expression of COMMD1 increased, and the expression of ATP7A and ATP7B decreased (Fig. 5G, I). In summary, the above data show that triptolide interferes with intracellular copper homeostasis by inhibiting the ubiquitination and degradation of COMMD1 by XIAP and subsequently downregulating the expression of ATP7A and ATP7B.

### Triptolide inhibits cervical cancer growth in vivo through cuproptosis

**Fig. 6:**
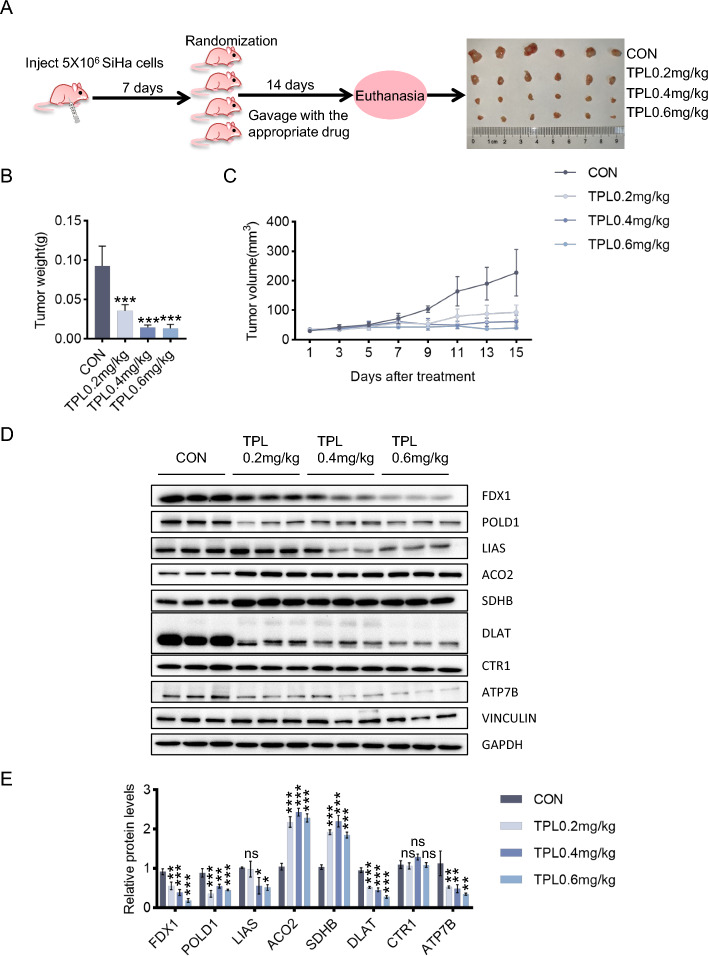
Triptolide inhibits cervical cancer growth in vivo through cuproptosis. **A** Establishment of a xenograft model in nude mice and an experimental schematic diagram. The tumor tissues from four rows were taken from the control, low-dose triptolide (0.2 mg/kg), middle-dose triptolide (0.4 mg/kg), and high-dose triptolide (0.6 mg/kg) groups. **B** Tumor weights were measured after the mice were euthanized. **C** Tumor volume was assessed every 2 days. **D**, **E** Protein content of tumor tissues. (*p < 0.05, **p < 0.01, ***p < 0.001 versus the CON group; ns, not significant.)

To explore triptolide suppression of the growth of cervical cancer in vivo, SiHa cells were used to establish a subcutaneous cervical cancer xenotransplantation model in mice. The tumors were removed after the mice were sacrificed (Fig. 6A). The tumor weights (Fig. 6B) and volumes (Fig. 6C) of the four groups were compared. Triptolide dramatically reduced tumor growth compared with that in the control group, and all three doses had anticancer effects. This result was also confirmed by immunohistochemistry staining of Ki67 (Additional file 1: Fig. S4A). The livers and kidneys were embedded in paraffin and stained with hematoxylin and eosin (Additional file 1: Fig. S4C). As expected, neither nephrotoxicity nor hepatotoxicity was observed in the four groups, indicating that this dose of triptolide has no serious toxicity or side effects on organs. In addition, no overt weight loss was observed in the triptolide treatment groups compared with the control group (Additional file 1: Fig. S4B).

To study whether triptolide induced cuproptosis of tumor cells in a mouse model of subcutaneous tumors, changes in the copper concentration and cuproptosis-related protein expression in the tumor tissues of the mice were evaluated. The copper content increased in the middle-dose and high-dose groups (Additional file 1: Fig. S4D). The results showed that CTR1 expression remained unchanged, while the protein expression of ATP7B decreased, the protein expression of FDX1, POLD1 and DLAT decreased, and that of LIAS did not significantly change in the low-dose group but decreased in the middle-dose and high-dose groups (Fig. 6D-E). These results were identical to the in vitro experimental results. However, the expression levels of the iron-sulfur cluster proteins ACO2 and SDHB increased, which is inconsistent with the in vitro experimental results (Fig. 6D-E).

## Discussion

The antitumor effects of copper ionophores, such as disulfiram (DSF) and elesclomol, which are mostly ROS-mediated cell death, autophagy and apoptosis, have been reported in many preclinical studies and clinical studies [27–29]. However, the antitumor mechanisms of copper ionophores have not yet been fully elucidated. A novel concept, cuproptosis, which is different from the known mechanisms of regulated cell death, has been proposed. The main target of cuproptosis is mitochondria, which are related to cell metabolism [5]. Although copper ionophore-mediated cancer cell death has been extensively studied, the role of cuproptosis in cancer has been less studied. The metabolic state of tumor cells determines their sensitivity to cuproptosis, and tumors with high aerobic respiration are more prone to cuproptosis. Using drugs to induce cuproptosis in these tumors as a cancer treatment method is feasible. In this study, HeLa and SiHa cell lines that rely on oxidative phosphorylation, were used to explore the pathway of cuproptosis; these cells have the objective conditions required for cuproptosis. Compared with that in other cervical cancer cell lines, such as Caski and C33A cells, the expression of FDX1 in HeLa and SiHa cells is greater, which means that cuproptosis is more likely to occur (Additional file 1: Fig. S2A) [5]. After treatment of the two cell lines with triptolide, the copper concentration in the HeLa and SiHa cells increased significantly, and the levels of cuproptosis-related proteins, such as FDX1, POLD1, LIAS, ACO2, SDHB, Lip-DLAT, Lip-DLST and DLAT, decreased in a dose-dependent manner. Moreover, the level of DLAT oligomer, which is toxic to cells, increased with increasing triptolide concentration [5]. These results suggest that triptolide induced cuproptosis in both cell lines. Furthermore, in this study, cuproptosis induced by triptolide in both cell lines was mitigated after pretreatment of HeLa and SiHa cells with the copper ion chelator TTM.

In vivo, the FDX1, POLD1, LIAS and DLAT levels were consistent with those in the in vitro results, and the levels of these proteins decreased in the triptolide administration group. The level of DLAT oligomers increased in the low- and middle-dose groups, but there was no significant change in the high-dose group, possibly because the expression of DLAT in the high-dose group was too low for oligomers of DLAT to form. Furthermore, loading buffer was added to all protein samples, which were then heated at 95 °C for 10 min for use in the western blot analysis. DLAT oligomers are linked by disulfide bonds, so this step may depolymerize some DLAT oligomers [5]. These two factors may explain why no changes were detected in the level of DLAT oligomers in the high-dose group. ACO2 is an iron-sulfur cluster protein that catalyzes the isomerization of citrate to isocitrate via cis-aconitate. SDHB is an iron-sulfur protein subunit of succinate dehydrogenase (SDH) that participates in complex II of the mitochondrial electron transport chain and is responsible for transferring electrons from succinate to ubiquinone. Both of these proteins are respiratory chain components. In this study, the level of ACO2 and SDHB increased in the triptolide administration group, which was inconsistent with the in vitro results. The metabolism of lipids, nucleic acids and amino acids is inhibited and the expression of components of the respiratory chain are downregulated in ATP7B knockout mice. However, lipid and nucleic acid metabolism were out of balance only in mice with a loss of ATP7B in hepatocytes, but the abundance of respiratory chain components and redox balance enzymes increased [30]. Thus, we inferred that the results of in vitro cell experiments are similar to those of ATP7B knockout mice, while the results of in vivo tumor experiments are similar to those of mice in which only ATP7B is lost in hepatocytes. We speculated that the increase in the expression levels of ACO2 and SDHB is related to the decrease in ATP7B in tumor tissues.

Both intracellular copper deficiency and copper overload can lead to disease, so maintaining copper homeostasis in cells is vital [31]. Cuproptosis is induced by intracellular copper accumulation. Therefore, to explore how triptolide induces an increase in intracellular copper levels and leads to cuproptosis, we first considered whether triptolide affects proteins involved in regulating copper homeostasis. Here, we revealed the key connection between XIAP and copper homeostasis in triptolide-treated cervical cancer cells. We demonstrated that triptolide upregulated COMMD1 expression and downregulated ATP7A and ATP7B expression by inhibiting XIAP, thereby promoting intracellular copper accumulation and subsequently inducing cuproptosis in cervical cancer cells. Previous research with similar results also revealed that the XIAP protein level greatly decreased as the copper concentration increased [32].

**Fig. 7:**
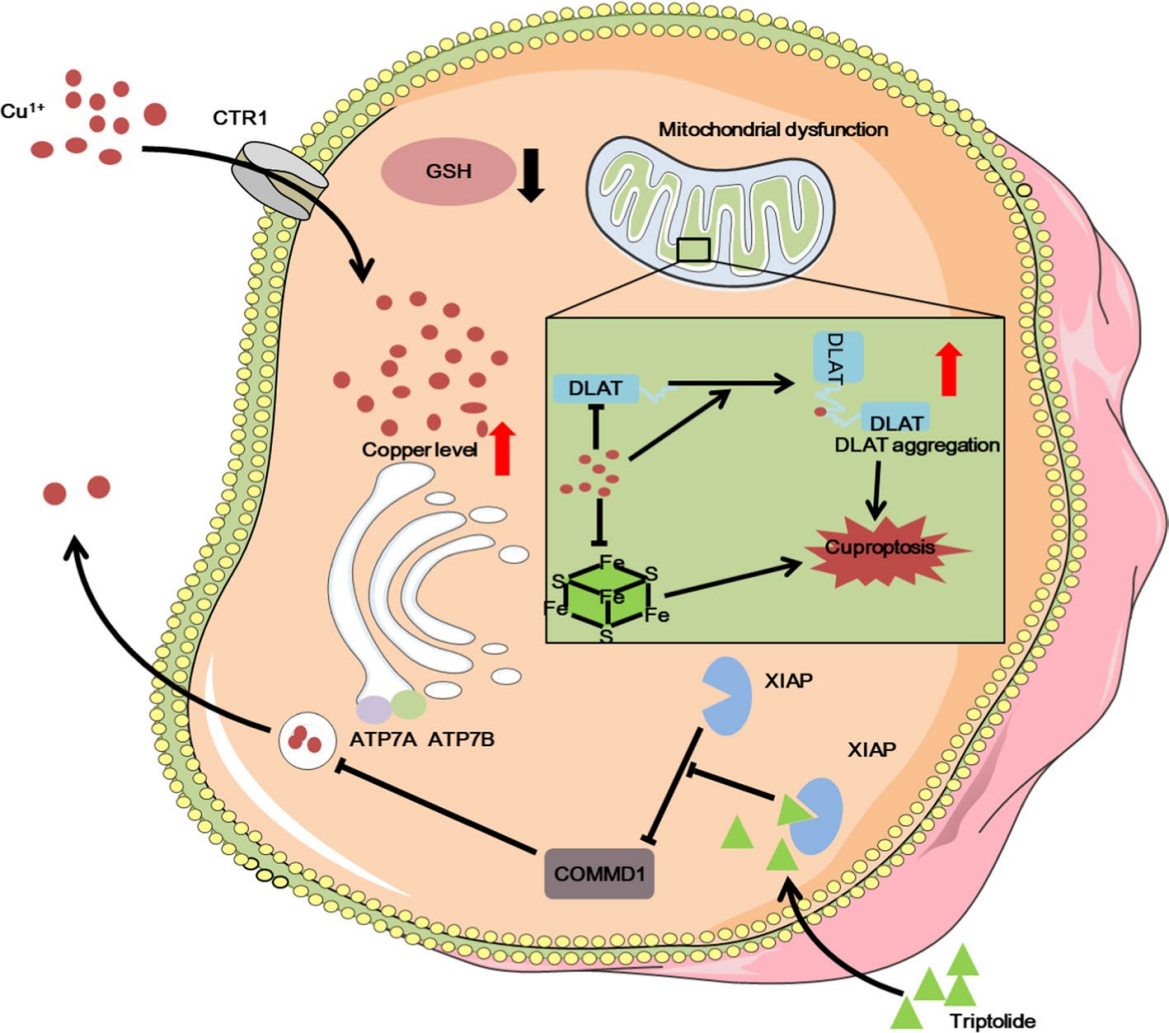
Schematic of the mechanisms by which triptolide induces cuproptosis in cervical cancer cells

Triptolide induces the apoptosis of tumor cells and plays an antitumor role [33, 34]. However, triptolide does not only induce the apoptosis of tumor cells. Treatment with triptolide and the caspase inhibitor Z-VAD-FMK or the caspase-3 inhibitor Z-DEV-FMK only weakens the lethality of triptolide to tumor cells but does not completely eliminate the lethality of triptolide [35]. This evidence shows that triptolide has many pathways to play an anti-tumor role (Fig. 3E-F). Indeed, many studies have reported that triptolide can regulate multiple pathways, such as the NFκB pathway, autophagy, pyroptosis, apoptosis, NLRP3 inflammasome pathway, Nrf2/HO-1 pathway and so on [12, 36–38]. Triptolide acts on XIAP, and overexpression of XIAP attenuates triptolide-induced cell death [39, 40]. XIAP is a major member of the IAP family and a powerful inhibitor of apoptosis that can directly inhibit caspases and regulate apoptosis via multiple mechanisms [22]. Triptolide can promote apoptosis by inhibiting XIAP in U937 cells, AML cells, KB cells,and gastric cancer cells [40–43]. Moreover, XIAP is also involved in the regulation of intracellular copper homeostasis. In this study, we reported a new antitumor mechanism by which triptolide promotes intracellular copper accumulation by regulating the XIAP/COMMD1 pathway and subsequently induces cuproptosis in cervical cancer cells (Fig. 7). These findings not only identify a new target of triptolide but also provide a theoretical reference for its clinical application in the treatment of cervical cancer.

Triptolide is not widely used in the clinic due to its hazardous side effects [44]. However, in this study, triptolide was given for a short time and at a low dose, which did not cause serious toxic effects in mice. Many new triptolide delivery systems have greatly improved the clinical application prospects of triptolide. For example, antibody-modified active targeting triptolide liposomes can specifically act on tumor cells and increase the drug concentration in tumor cells to enhance the therapeutic effect (45).

## Conclusions

In summary, we report a new antitumor mechanism of triptolide, which disrupted intracellular copper homeostasis and induced cuproptosis in cervical cancer cells by regulating the XIAP/COMMD1/ATP7A/B axis. These findings not only identify a new target of triptolide but also provide a theoretical reference for its clinical application in the treatment of cervical cancer.

### Supplementary Information

Additional file 1. Figure S1. Triptolide inhibits the proliferation and migration of cervical cancer cells.IC_50_ of triptolide in HeLa and SiHa cells.HeLa and SiHa cells were treated with 0, 20, 40 or 80 nM triptolide for 0, 24, 48 or 72 h.Results from the EdU cell proliferation assay of in HeLa and SiHa cells with or without triptolide treatment for 24 h. Scale bars: 100 μm.Wound healing assay of in HeLa and SiHa cells with or without triptolide treatment for 48 h. Scale bars: 50 μm.Transwell migration assay of in HeLa and SiHa cells with or without triptolide treatment. Scale bars: 100 μm.

Additional file 2. Figure S2. Triptolide increased the intracellular copper concentration in a time-dependent manner.FDX1 expression levels in HeLa, Caski, SiHa and C33A cells.Representative photographs of Cu^2+^ fluorescence in HeLa and SiHa cells treated with triptolide for 0, 12, 24, 36, or 48 h. Scale bars: 100 μm.

Additional file 3. Figure S3. Triptolide changed the morphology of cervical cancer cells.Representative electron microscopy images of HeLa and SiHa cells treated with or without triptolide for 48 h

Additional file 4. Figure S4. Triptolide had no serious toxicity or side effects in a nude mouse xenograft model.Ki67 expression levels in tumor tissues from the control group and middle-dose triptolide group.Mouse weights were measured every 2 days.H&E staining of livers and kidneys isolated from mice at 15 days after treatment. Scale bars: 100 μm.Representative images of copper salt-stained tumor tissues

Additional file 5. Figure S5. Triptolide inhibits the proteasomal degradation of COMMD1.Western blot analysis of XIAP and COMMD1 with or without 2 μM MG132 treatment for 24 h and 80 nM triptolide for 48 h in HeLa cells.Western blot analysis of XIAP and COMMD1 with or without 2 μM MG132 treatment and 40 nM triptolide for 48 h for 24 h in SiHa cells. It is possible that there are skimmed milk powder particles in the blocking solution, which leads to the existence of black particles in the background of COMMD1 lane.

## Data Availability

All data generated or analysed during the current study are available from the corresponding author on reasonable request.

## Abbreviations

TTM: Tetrathiomolybdate
TPL: Triptolide
MTT: Thiazolyl blue tetrazolium bromide
Fer-1: Ferrostatin-1
Z-VAD: Z-VAD (OMe)-FMK
3-MA: 3-Methyladenine
Nec-1: Necrostatin-1
NAC: Acetylcysteine
DMSO: Dimethyl sulfoxide
GSH: Glutathione
ATCC: American Tissue Culture Collection
OD: Optical density
IC_50_: Half maximal inhibitory concentration
PBS: Phosphate buffered saline
FBS: Fetal bovine serum
ICP-MS: Inductively coupled plasma mass spectrometry
DSF: Disulfiram

## References

[1] Dixon SJ, Lemberg KM, Lamprecht MR, Skouta R, Zaitsev EM, Gleason CE, et al. Ferroptosis: an iron-dependent form of nonapoptotic cell death. Cell. 2012;149(5):1060–72. 22632970 10.1016/j.cell.2012.03.042 PMC3367386

[2] Du W, Gu M, Hu M, Pinchi P, Chen W, Ryan M, et al. Lysosomal Zn (2+) release triggers rapid, mitochondria-mediated, non-apoptotic cell death in metastatic melanoma. Cell Rep. 2021;37(3): 109848. 34686351 10.1016/j.celrep.2021.109848 PMC8559338

[3] Zhao X, Liu Y, Zhu G, Liang Y, Liu B, Wu Y, et al. SIRT1 downregulation mediated Manganese-induced neuronal apoptosis through activation of FOXO3a-Bim/PUMA axis. Sci Total Environ. 2019;646:1047–55. 30235590 10.1016/j.scitotenv.2018.07.363

[4] Bai S, Lan Y, Fu S, Cheng H, Lu Z, Liu G. Connecting calcium-based nanomaterials and cancer: from diagnosis to therapy. Nanomicro Lett. 2022;14(1):145. 35849180 10.1007/s40820-022-00894-6 PMC9294135

[5] Tsvetkov P, Coy S, Petrova B, Dreishpoon M, Verma A, Abdusamad M, et al. Copper induces cell death by targeting lipoylated TCA cycle proteins. Science. 2022;375(6586):1254–61. 35298263 10.1126/science.abf0529 PMC9273333

[6] AbdulHussein AH, Al-Taee MM, Radih ZA, Aljuboory DS, Mohammed ZQ, Hashesh TS, et al. Mechanisms of cancer cell death induction by triptolide. BioFactors. 2023. 10.1002/biof.1944. 36876465 10.1002/biof.1944

[7] He X, Wang N, Zhang Y, Huang X, Wang Y. The therapeutic potential of natural products for treating pancreatic cancer. Front Pharmacol. 2022;13:1051952. 36408249 10.3389/fphar.2022.1051952 PMC9666876

[8] Li H, Takai N, Yuge A, Furukawa Y, Tsuno A, Tsukamoto Y, et al. Novel target genes responsive to the anti-growth activity of triptolide in endometrial and ovarian cancer cells. Cancer Lett. 2010;297(2):198–206. 20547442 10.1016/j.canlet.2010.05.012

[9] Li JX, Shi JF, Wu YH, Xu HT, Fu CM, Zhang JM. Mechanisms and application of triptolide against breast cancer. Zhongguo Zhong Yao Za Zhi. 2021;46(13):3249–56. 34396744 10.19540/j.cnki.cjcmm.20210225.601

[10] Wu X, Wang J, Li B, Gong M, Cao C, Song L, et al. Chlorogenic acid, rutin, and quercetin from Lysimachia christinae alleviate triptolide-induced multi-organ injury in vivo by modulating immunity and AKT/mTOR signal pathway to inhibit ferroptosis and apoptosis. Toxicol Appl Pharmacol. 2023;467: 116479. 36963520 10.1016/j.taap.2023.116479

[11] Ren T, Tang YJ, Wang MF, Wang HS, Liu Y, Qian X, et al. Triptolide induces apoptosis through the calcium/calmodulin-dependent protein kinase kinasebeta/AMP-activated protein kinase signaling pathway in non-small cell lung cancer cells. Oncol Rep. 2020;44(5):2288–96. 33000264 10.3892/or.2020.7763

[12] Sanadgol N, Golab F, Mostafaie A, Mehdizadeh M, Khalseh R, Mahmoudi M, et al. Low, but not high, dose triptolide controls neuroinflammation and improves behavioral deficits in toxic model of multiple sclerosis by dampening of NF-kappaB activation and acceleration of intrinsic myelin repair. Toxicol Appl Pharmacol. 2018;342:86–98. 29407366 10.1016/j.taap.2018.01.023

[13] Xie J, Yang Y, Gao Y, He J. Cuproptosis: mechanisms and links with cancers. Mol Cancer. 2023;22(1):46. 36882769 10.1186/s12943-023-01732-y PMC9990368

[14] Van Hee VF, Perez-Escuredo J, Cacace A, Copetti T, Sonveaux P. Lactate does not activate NF-kappaB in oxidative tumor cells. Front Pharmacol. 2015;6:228. 26528183 10.3389/fphar.2015.00228 PMC4602127

[15] Bray F, Ferlay J, Soerjomataram I, Siegel RL, Torre LA, Jemal A. Global cancer statistics 2018: GLOBOCAN estimates of incidence and mortality worldwide for 36 cancers in 185 countries. CA Cancer J Clin. 2018;68(6):394–424. 30207593 10.3322/caac.21492

[16] Siegel RL, Giaquinto AN, Jemal A. Cancer statistics, 2024. CA Cancer J Clin. 2024;74(1):12–49. 38230766 10.3322/caac.21820

[17] Liu Y, Yang X, Gan J, Chen S, Xiao ZX, Cao Y. CB-Dock2: improved protein-ligand blind docking by integrating cavity detection, docking and homologous template fitting. Nucleic Acids Res. 2022;50(W1):W159–64. 35609983 10.1093/nar/gkac394 PMC9252749

[18] Yang F, Pei R, Zhang Z, Liao J, Yu W, Qiao N, et al. Copper induces oxidative stress and apoptosis through mitochondria-mediated pathway in chicken hepatocytes. Toxicol In Vitro. 2019;54:310–6. 30389602 10.1016/j.tiv.2018.10.017

[19] Chen J, Jiang Y, Shi H, Peng Y, Fan X, Li C. The molecular mechanisms of copper metabolism and its roles in human diseases. Pflugers Arch. 2020;472(10):1415–29. 32506322 10.1007/s00424-020-02412-2

[20] Jin J, Ma M, Shi S, Wang J, Xiao P, Yu HF, et al. Copper enhances genotoxic drug resistance via ATOX1 activated DNA damage repair. Cancer Lett. 2022;536: 215651. 35315340 10.1016/j.canlet.2022.215651

[21] Chen L, Min J, Wang F. Copper homeostasis and cuproptosis in health and disease. Signal Transduct Target Ther. 2022;7(1):378. 36414625 10.1038/s41392-022-01229-y PMC9681860

[22] Hanifeh M, Ataei F. XIAP as a multifaceted molecule in cellular signaling. Apoptosis. 2022;27(7–8):441–53. 35661061 10.1007/s10495-022-01734-z

[23] Mufti AR, Burstein E, Duckett CS. XIAP: cell death regulation meets copper homeostasis. Arch Biochem Biophys. 2007;463(2):168–74. 17382285 10.1016/j.abb.2007.01.033 PMC1986780

[24] Burstein E, Ganesh L, Dick RD, van De Sluis B, Wilkinson JC, Klomp LW, et al. A novel role for XIAP in copper homeostasis through regulation of MURR1. EMBO J. 2004;23(1):244–54. 14685266 10.1038/sj.emboj.7600031 PMC1271669

[25] Liu Y, Zhao ZH, Wang T, Yao JY, Wei WQ, Su LH, et al. Lead exposure disturbs ATP7B-mediated copper export from brain barrier cells by inhibiting XIAP-regulated COMMD1 protein degradation. Ecotoxicol Environ Saf. 2023;256: 114861. 37027943 10.1016/j.ecoenv.2023.114861

[26] Materia S, Cater MA, Klomp LW, Mercer JF, La Fontaine S. Clusterin and COMMD1 independently regulate degradation of the mammalian copper ATPases ATP7A and ATP7B. J Biol Chem. 2012;287(4):2485–99. 22130675 10.1074/jbc.M111.302216 PMC3268409

[27] Lu C, Li X, Ren Y, Zhang X. Disulfiram: a novel repurposed drug for cancer therapy. Cancer Chemother Pharmacol. 2021;87(2):159–72. 33426580 10.1007/s00280-020-04216-8

[28] O’Day SJ, Eggermont AM, Chiarion-Sileni V, Kefford R, Grob JJ, Mortier L, et al. Final results of phase III SYMMETRY study: randomized, double-blind trial of elesclomol plus paclitaxel versus paclitaxel alone as treatment for chemotherapy-naive patients with advanced melanoma. J Clin Oncol. 2013;31(9):1211–8. 23401447 10.1200/JCO.2012.44.5585

[29] Li H, Wang J, Wu C, Wang L, Chen ZS, Cui W. The combination of disulfiram and copper for cancer treatment. Drug Discov Today. 2020;25(6):1099–108. 32320854 10.1016/j.drudis.2020.04.003

[30] Muchenditsi A, Talbot CC Jr, Gottlieb A, Yang H, Kang B, Boronina T, et al. Systemic deletion of Atp7b modifies the hepatocytes’ response to copper overload in the mouse models of Wilson disease. Sci Rep. 2021;11(1):5659. 33707579 10.1038/s41598-021-84894-3 PMC7952580

[31] Wang Z, Jin D, Zhou S, Dong N, Ji Y, An P, et al. Regulatory roles of copper metabolism and cuproptosis in human cancers. Front Oncol. 2023;13:1123420. 37035162 10.3389/fonc.2023.1123420 PMC10076572

[32] Mufti AR, Burstein E, Csomos RA, Graf PC, Wilkinson JC, Dick RD, et al. XIAP Is a copper binding protein deregulated in Wilson’s disease and other copper toxicosis disorders. Mol Cell. 2006;21(6):775–85. 16543147 10.1016/j.molcel.2006.01.033

[33] Zheng Z, Yan G, Xi N, Xu X, Zeng Q, Wu Y, et al. Triptolide induces apoptosis and autophagy in cutaneous squamous cell carcinoma via Akt/mTOR pathway. Anticancer Agents Med Chem. 2023;23(13):1596–604. 37056067 10.2174/1871520623666230413130417

[34] Qin W, Li S, Miao Y, Shi Q, Wang Y, Li J, et al. Triptolide induces mitochondrial apoptosis through modulating dual specificity phosphatase 1/mitogen-activated protein kinases cascade in osteosarcoma cells. Neoplasma. 2018;65(1):21–33. 29322785 10.4149/neo_2018_170109N16

[35] Park SW, Kim YI. Triptolide induces apoptosis of PMA-treated THP-1 cells through activation of caspases, inhibition of NF-kappaB and activation of MAPKs. Int J Oncol. 2013;43(4):1169–75. 23900299 10.3892/ijo.2013.2033

[36] Huo J, Yu Q, Zhang Y, Liu K, Hsiao CD, Jiang Z, et al. Triptolide-induced hepatotoxicity via apoptosis and autophagy in zebrafish. J Appl Toxicol. 2019;39(11):1532–40. 31321794 10.1002/jat.3837

[37] Zhang HR, Li YP, Shi ZJ, Liang QQ, Chen SY, You YP, et al. Triptolide induces PANoptosis in macrophages and causes organ injury in mice. Apoptosis. 2023;28(11–12):1646–65. 37702860 10.1007/s10495-023-01886-6

[38] Lv C, Cheng T, Zhang B, Sun K, Lu K. Triptolide protects against podocyte injury in diabetic nephropathy by activating the Nrf2/HO-1 pathway and inhibiting the NLRP3 inflammasome pathway. Ren Fail. 2023;45(1):2165103. 36938748 10.1080/0886022X.2023.2165103 PMC10035962

[39] Carter BZ, Mak DH, Schober WD, McQueen T, Harris D, Estrov Z, et al. Triptolide induces caspase-dependent cell death mediated via the mitochondrial pathway in leukemic cells. Blood. 2006;108(2):630–7. 16556893 10.1182/blood-2005-09-3898 PMC1895484

[40] Carter BZ, Mak DH, Schober WD, Dietrich MF, Pinilla C, Vassilev LT, et al. Triptolide sensitizes AML cells to TRAIL-induced apoptosis via decrease of XIAP and p53-mediated increase of DR5. Blood. 2008;111(7):3742–50. 18187663 10.1182/blood-2007-05-091504 PMC2275030

[41] Chen YW, Lin GJ, Chuang YP, Chia WT, Hueng DY, Lin CK, et al. Triptolide circumvents drug-resistant effect and enhances 5-fluorouracil antitumor effect on KB cells. Anticancer Drugs. 2010;21(5):502–13. 20154595 10.1097/CAD.0b013e328337337c

[42] Wang BY, Cao J, Chen JW, Liu QY. Triptolide induces apoptosis of gastric cancer cells via inhibiting the overexpression of MDM2. Med Oncol. 2014;31(11):270. 25280518 10.1007/s12032-014-0270-7

[43] Choi YJ, Kim TG, Kim YH, Lee SH, Kwon YK, Suh SI, et al. Immunosuppressant PG490 (triptolide) induces apoptosis through the activation of caspase-3 and down-regulation of XIAP in U937 cells. Biochem Pharmacol. 2003;66(2):273–80. 12826269 10.1016/S0006-2952(03)00282-X

[44] Song J, He GN, Dai L. A comprehensive review on celastrol, triptolide and triptonide: Insights on their pharmacological activity, toxicity, combination therapy, new dosage form and novel drug delivery routes. Biomed Pharmacother. 2023;162: 114705. 37062220 10.1016/j.biopha.2023.114705

[45] Wickens JM, Alsaab HO, Kesharwani P, Bhise K, Amin M, Tekade RK, et al. Recent advances in hyaluronic acid-decorated nanocarriers for targeted cancer therapy. Drug Discov Today. 2017;22(4):665–80. 28017836 10.1016/j.drudis.2016.12.009 PMC5413407

