# Supplementary material for: Triptolide-induced cuproptosis is a novel antitumor strategy for the treatment of cervical cancer
Source: Cell Mol Biol Lett. 2024 Aug 28;29:113. doi: 10.1186/s11658-024-00623-4 (PMC11360305; doi:10.1186/s11658-024-00623-4)
Supplement: Supplementary file 1 — Additional file 1. Figure S1. Triptolide inhibits the proliferation and migration of cervical cancer cells.IC50 of triptolide in HeLa and SiHa cells.HeLa and SiHa cells were treated with 0, 20, 40 or 80 nM triptolide for 0, 24, 48 or 72 h.Results from the EdU cell proliferation assay of in HeLa and SiHa cells with or without triptolide treatment for 24 h. Scale bars: 100 μm.Wound healing assay of in HeLa and SiHa cells with or without triptolide treatment for 48 h. Scale bars: 50 μm.Transwell migration assay of in HeLa and SiHa cells with or without triptolide treatment. Scale bars: 100 μm. [file 11658_2024_623_MOESM1_ESM.pptx]

## Slide 1
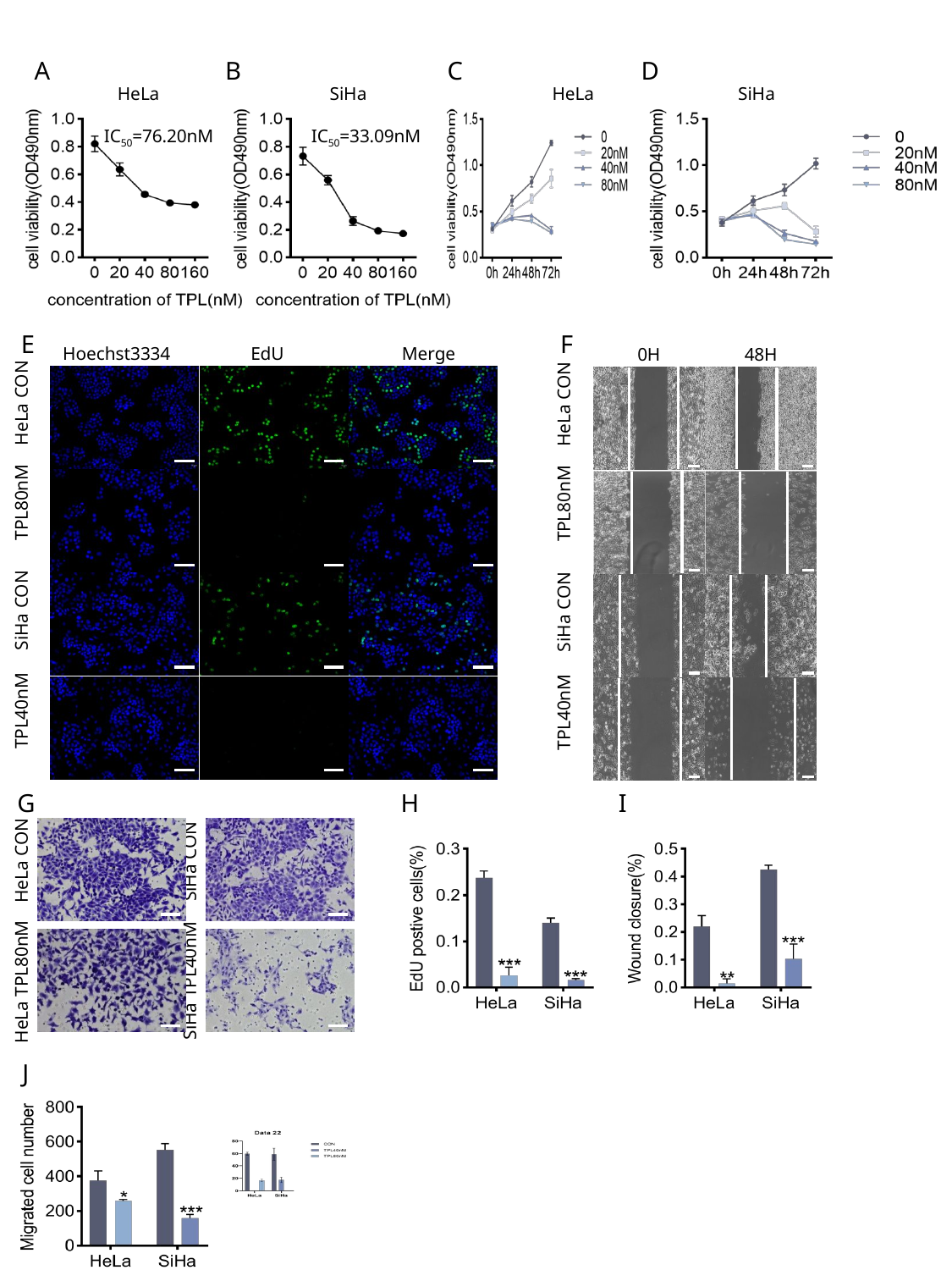

A
B
C
D
HeLa
SiHa
HeLa
SiHa
IC50=76.20nM
IC50=33.09nM
F
0H
48H
HeLa CON
TPL80nM
SiHa CON
TPL40nM
E
Hoechst33342
EdU
Merge
HeLa CON
 TPL80nM
SiHa CON
TPL40nM
G
H
I
HeLa CON
SiHa CON
HeLa TPL80nM
SiHa TPL40nM
J
