# Supplementary material for: Triptolide-induced cuproptosis is a novel antitumor strategy for the treatment of cervical cancer
Source: Cell Mol Biol Lett. 2024 Aug 28;29:113. doi: 10.1186/s11658-024-00623-4 (PMC11360305; doi:10.1186/s11658-024-00623-4)
Supplement: Supplementary file 2 — Additional file 2. Figure S2. Triptolide increased the intracellular copper concentration in a time-dependent manner.FDX1 expression levels in HeLa, Caski, SiHa and C33A cells.Representative photographs of Cu2+ fluorescence in HeLa and SiHa cells treated with triptolide for 0, 12, 24, 36, or 48 h. Scale bars: 100 μm. [file 11658_2024_623_MOESM2_ESM.pptx]

## Slide 1
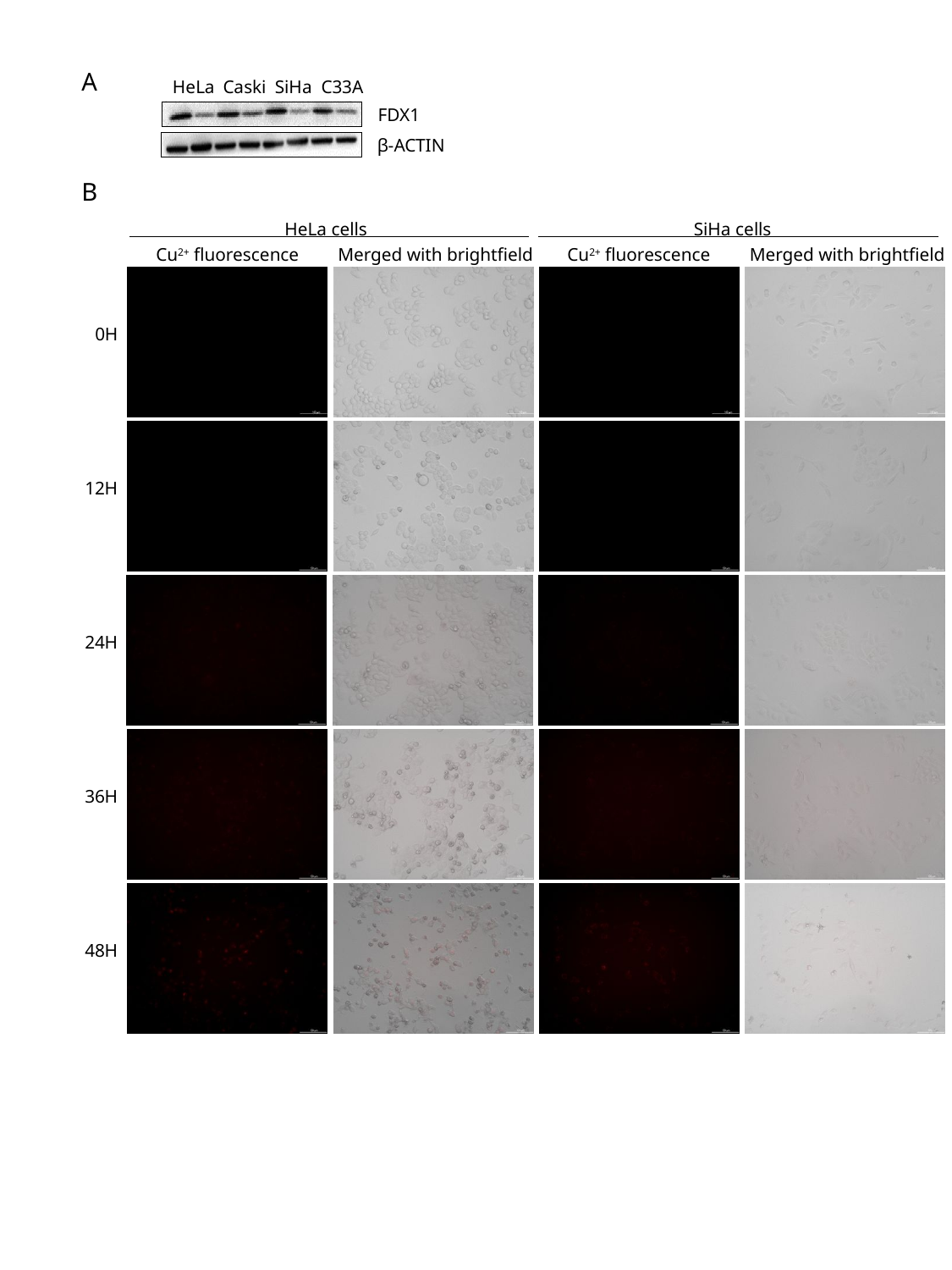

A
HeLa
Caski
SiHa
C33A
FDX1
β-ACTIN
B
HeLa cells
SiHa cells
Cu2+ fluorescence
Merged with brightfield
Cu2+ fluorescence
Merged with brightfield
0H
12H
24H
36H
48H
