# Supplementary figures and images for: Triptolide-induced cuproptosis is a novel antitumor strategy for the treatment of cervical cancer
Source: Cell Mol Biol Lett. 2024 Aug 28;29:113. doi: 10.1186/s11658-024-00623-4 (PMC11360305; doi:10.1186/s11658-024-00623-4)

## Slide 1
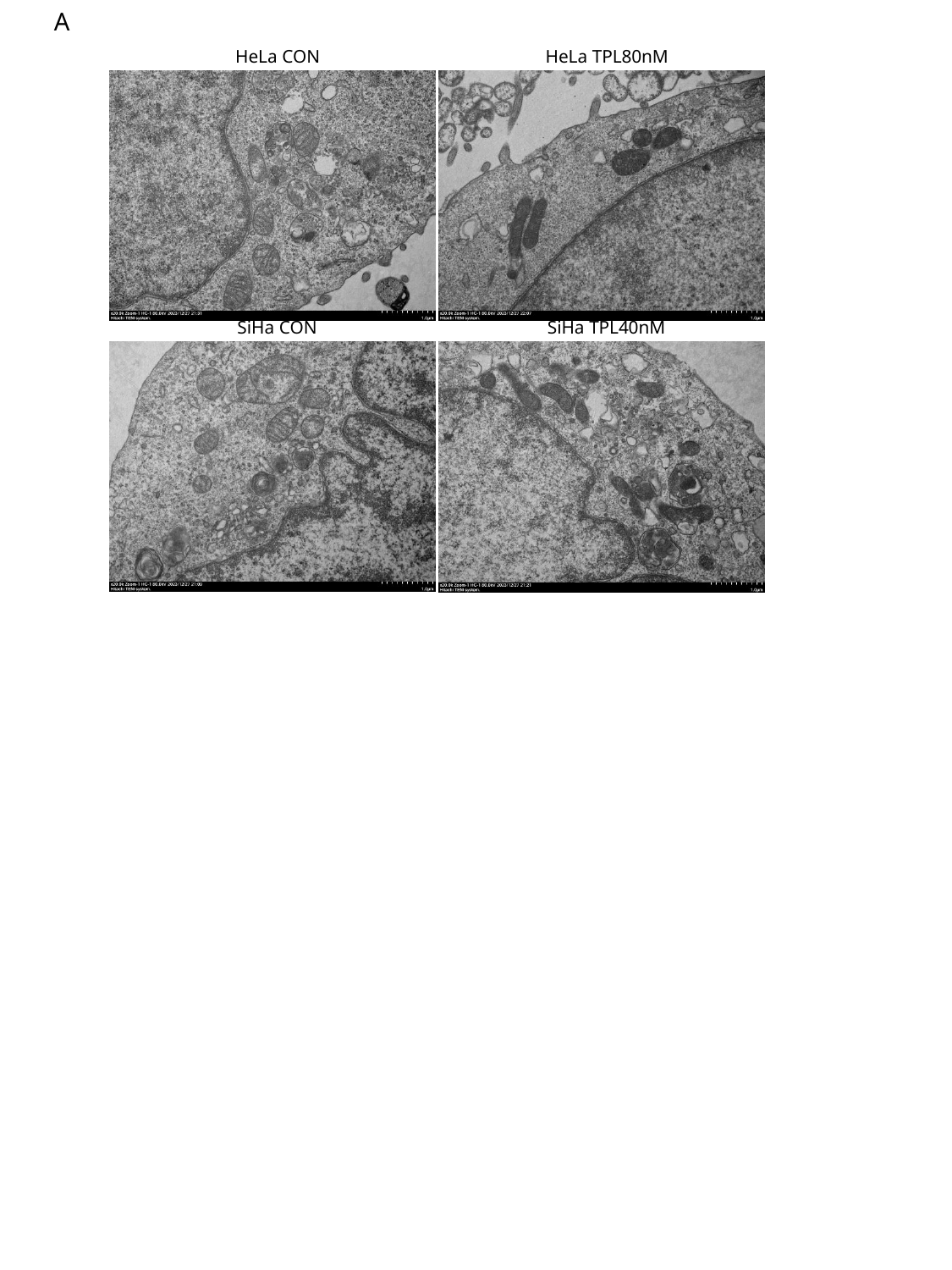

HeLa TPL80nM
A
HeLa CON
SiHa TPL40nM
SiHa CON

Supplement: Supplementary file 3 — Additional file 3. Figure S3. Triptolide changed the morphology of cervical cancer cells.Representative electron microscopy images of HeLa and SiHa cells treated with or without triptolide for 48 h [file 11658_2024_623_MOESM3_ESM.pptx]

## Slide 1
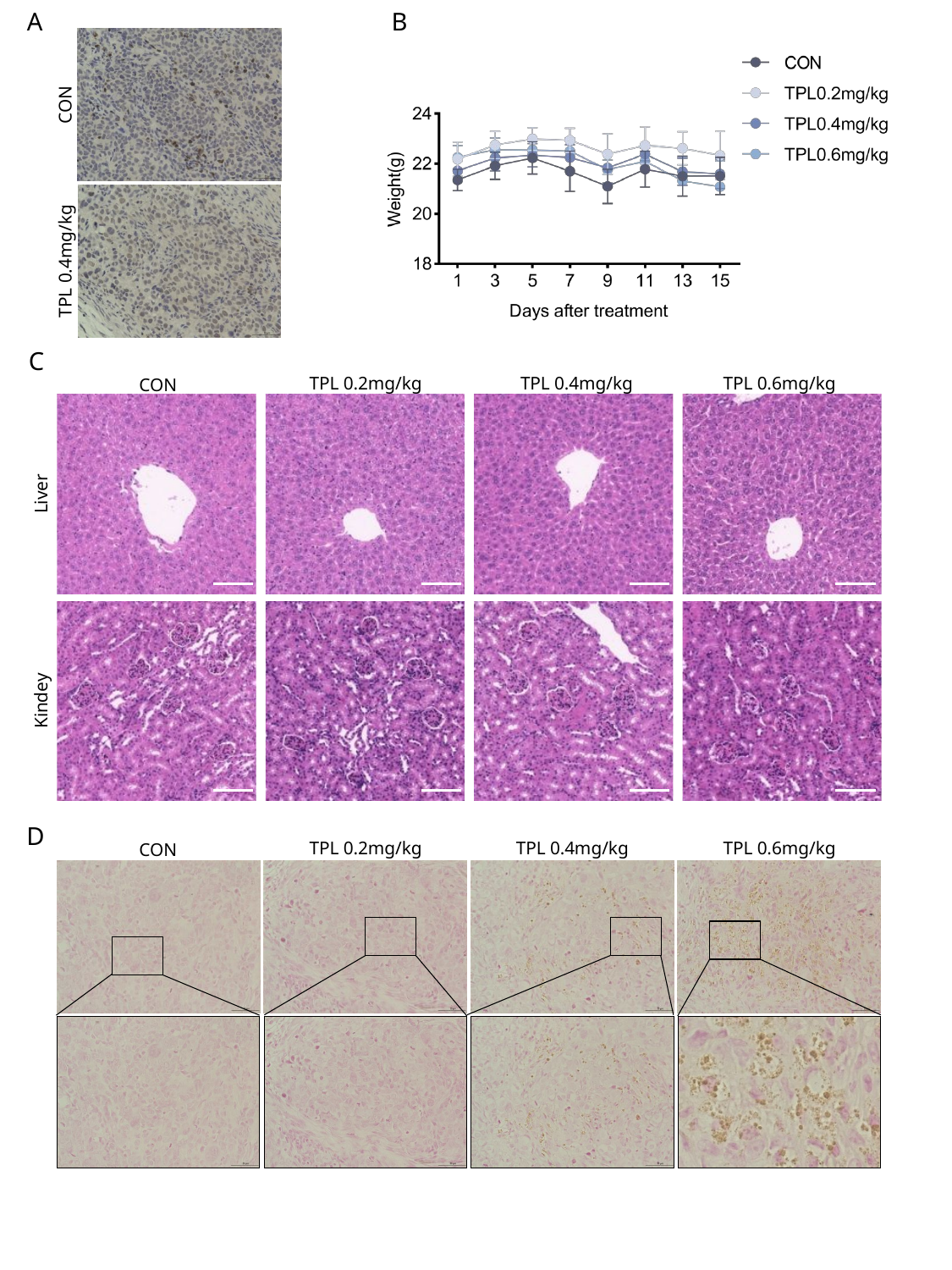

A
B
CON
TPL 0.4mg/kg
C
TPL 0.2mg/kg
TPL 0.4mg/kg
TPL 0.6mg/kg
CON
Liver
Kindey
D
TPL 0.2mg/kg
TPL 0.4mg/kg
TPL 0.6mg/kg
CON

Supplement: Supplementary file 4 — Additional file 4. Figure S4. Triptolide had no serious toxicity or side effects in a nude mouse xenograft model.Ki67 expression levels in tumor tissues from the control group and middle-dose triptolide group.Mouse weights were measured every 2 days.H&E staining of livers and kidneys isolated from mice at 15 days after treatment. Scale bars: 100 μm.Representative images of copper salt-stained tumor tissues [file 11658_2024_623_MOESM4_ESM.pptx]
