# Supplementary material for: Triptolide-induced cuproptosis is a novel antitumor strategy for the treatment of cervical cancer
Source: Cell Mol Biol Lett. 2024 Aug 28;29:113. doi: 10.1186/s11658-024-00623-4 (PMC11360305; doi:10.1186/s11658-024-00623-4)
Supplement: Supplementary file 5 — Additional file 5. Figure S5. Triptolide inhibits the proteasomal degradation of COMMD1.Western blot analysis of XIAP and COMMD1 with or without 2 μM MG132 treatment for 24 h and 80 nM triptolide for 48 h in HeLa cells.Western blot analysis of XIAP and COMMD1 with or without 2 μM MG132 treatment and 40 nM triptolide for 48 h for 24 h in SiHa cells. It is possible that there are skimmed milk powder particles in the blocking solution, which leads to the existence of black particles in the background of COMMD1 lane. [file 11658_2024_623_MOESM5_ESM.pptx]

## Slide 1
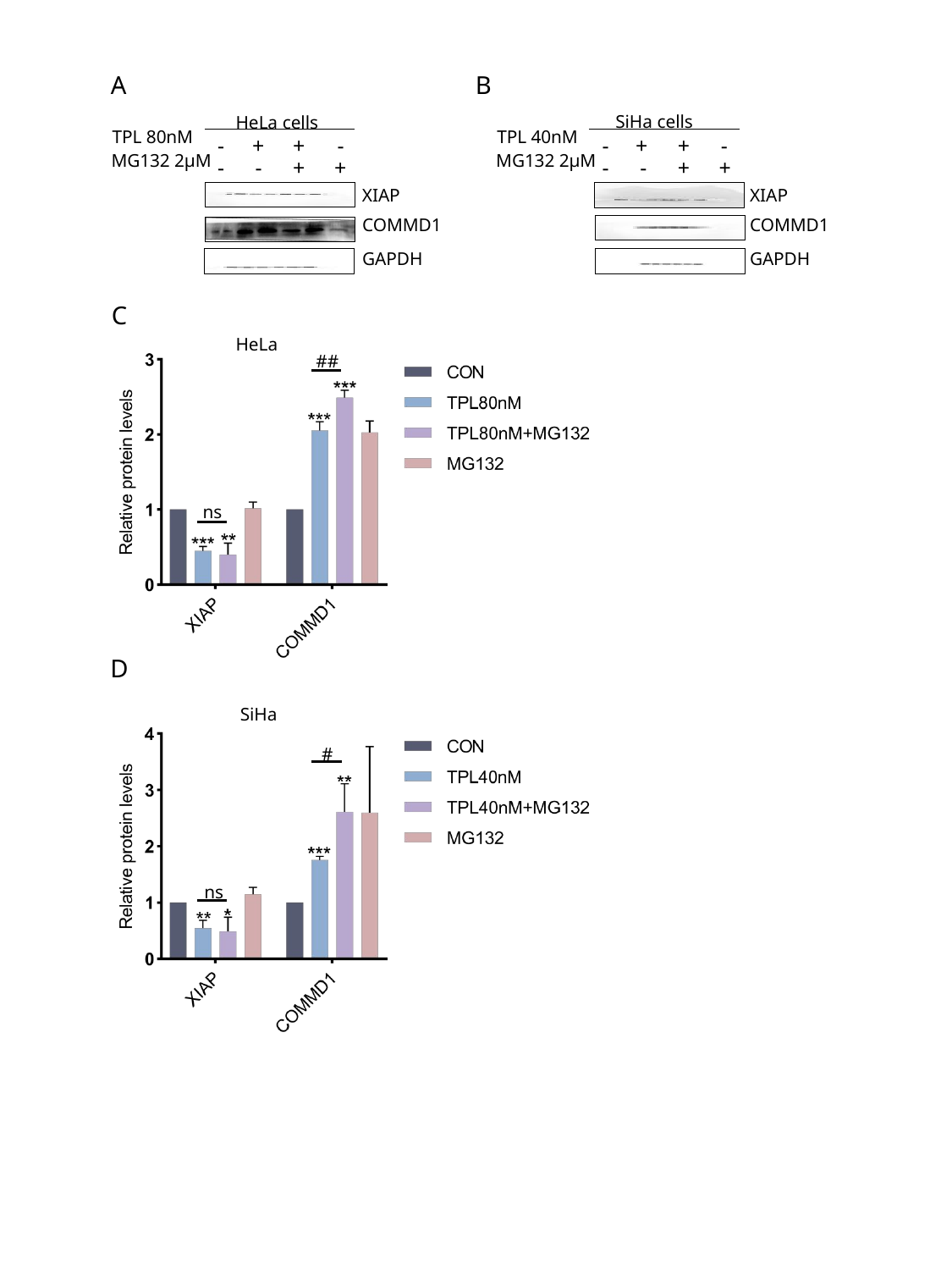

A
B
SiHa cells
HeLa cells
TPL 80nM
-
+
+
-
MG132 2μM
-
-
+
+
TPL 40nM
-
+
+
-
MG132 2μM
-
-
+
+
XIAP
XIAP
COMMD1
COMMD1
GAPDH
GAPDH
C
HeLa
##
ns
D
SiHa
#
ns
